# Quantitative evaluation of iron chelator effects on central motor and sensory tracts in superficial siderosis

**DOI:** 10.3389/fneur.2026.1734951

**Published:** 2026-02-18

**Authors:** Ryo Iwase, Nobuo Sanjo, Tadashi Kanouchi, Yurie Nose, Miho Akaza, Motonori Hashimoto, Satoru Egawa, Toshitaka Yoshii, Takanori Yokota

**Affiliations:** 1 Department of Neurology and Neurological Sciences, Graduate School of Medical and Dental Sciences, Institute of Science Tokyo, Tokyo, Japan; 2 Department of Laboratory Medicine, Graduate School of Medical and Dental Sciences, Institute of Science Tokyo, Tokyo, Japan; 3 Department of Respiratory and Nervous System Science, Institute of Science Tokyo, Tokyo, Japan; 4 Department of Orthopaedic Surgery, Graduate School of Medical and Dental Sciences, Institute of Science Tokyo, Tokyo, Japan

**Keywords:** central motor tracts, central sensory tracts, iron chelator, quantitative evaluation, superficial siderosis

## Abstract

**Background:**

Superficial siderosis (SS) is a rare neurodegenerative disease characterized by hemosiderin deposition in the central nervous system, leading to progressive neuronal degeneration. Although myelopathy is a main clinical manifestation in SS, objective biomarkers for monitoring disease progression and treatment remain unclarified. We assessed electrophysiology of central motor and sensory conduction in patients with SS before and after iron chelator administration.

**Methods:**

In this controlled trial, we evaluated 12 patients with SS (mean follow-up: 33.6 months [range: 18.9–36.1]), who underwent dural repair, and 9 with multiple sclerosis as controls. Transcranial magnetic stimulation (TMS) and somatosensory evoked potentials (SEPs) were used to assess motor and sensory conduction, respectively. Eight patients with SS received oral iron chelator deferiprone (1,500 mg/day), whereas four underwent surgical repair without iron chelation. Primary outcomes were changes in central motor conduction time (CMCT), motor evoked potential (MEP) amplitude, and central sensory conduction time (CCT).

**Results:**

At baseline, 92 and 100% of patients with SS exhibited prolonged CMCT and CCT, respectively. Prolonged conduction in patients with SS was less severe than in those with multiple sclerosis (MS). Whereas MEP amplitude and the amplitude ratio of MEP to CMAP were relatively preserved in patients with SS. After 36-month iron chelator administration, statistically significant improvement of central motor conduction time was gained in patients with SS.

**Conclusion:**

TMS and SEP are sensitive tools for evaluating central conduction impairment in SS. The conduction abnormality, likely caused by a different pathomechanism from that in MS, is partially recoverable by iron chelator administration. CMCT improvement demonstrates partial reversibility of impaired motor pathway and supports the therapeutic potential of iron chelator therapy in SS.

## 1 Introduction

Superficial siderosis (SS) is a rare neurodegenerative disease primarily characterized by cerebellar ataxia, sensorineural hearing loss, and myelopathy (1–5). SS results from chronic or intermittent micro bleeding of small intradural vessels into the subarachnoid space, leading to the release of neurotoxic heme degradation products and their accumulation in the brain and spinal cord (1, 2, 5, 6). These processes trigger oxidative stress, ferroptosis, inflammation, and subsequent neurotoxicity, ultimately causing myelopathy (2, 6). Myelopathy, affecting approximately 53% of patients, typically presents with hyperreflexia, spastic gait, and urinary dysfunction (7).

Although prior studies have demonstrated the clinical efficacy of blood–brain barrier-permeable oral iron chelators (4, 8), their impact on myelopathy remains unexplored. To objectively assess spinal tract impairment, we aimed to evaluate the functional status of the corticospinal and sensory pathways in the nervous system of patients with SS.

## 2 Methods

This was a non-randomized controlled trial conducted in accordance with the principles of the Declaration of Helsinki (2013) (9). The study protocol was approved by the Institutional Ethics Committee of the Institute of Science Tokyo Hospital. Informed consent was obtained from each patient.

We recruited patients from October 6, 2010 to April 18, 2022. Of the 62 patients with SS who visited the Institute of Science Tokyo Hospital, 12 consented to participate. Participants underwent surgical closure of their dural defects and were divided into two groups based on the treatment approach (Supplementary Table 1). The chelator administration (CA) group comprised eight patients who received deferiprone (1,500 mg/day) for 36 months, whereas the non-administration control (NC) group included the remaining four patients. Nine participants with multiple sclerosis (MS) served as controls to represent demyelinating diseases (Supplementary Figure 1).

### 2.1 Electrophysiological assessments

Electrophysiological assessments were conducted at baseline and at the end of the study period.

#### 2.1.1 Transcranial magnetic stimulation

Transcranial magnetic stimulation (TMS) to the foot area of the motor cortex was performed using a Magstim 200 with a single 90 mm standard circular coil (Magstim Company, Whitland, Dyfed, UK). The posterior edge of the coil was placed over about 2 cm posterior to Cz (international 10–20 system for electroencephalogram). The stimulation was delivered at 100% maximum stimulator output with the direction of induced electrical current perpendicular to the interhemispheric cleavage from the medial to the lateral side of the target hemisphere. Motor evoked potentials (MEPs) were recorded from the Ag-AgCl surface cup electrodes placed on the bilateral abductor hallucis (AH) muscles using the belly-tendon method under slight voluntary muscle contraction. We also evaluated the resting motor threshold (rMT), which was determined as the lowest stimulus intensity of TMS at rest to elicit MEPs of >50 μV in 50% of 10 consecutive trials (10).

Compound muscle action potentials (CMAPs) and 20 consecutive F waves were also recorded from bilateral AH muscles through supramaximal electrical stimulation with 0.5 ms of square pulse at the ankle to evaluate central and peripheral conduction parameters: central motor conduction time (CMCT), distal latency of the CMAP (M), the shortest latency of F waves (F), peripheral motor conduction time (PMCT), and cauda equina conduction time (CECT). All responses (MEPs, CMAPs, and F waves) were amplified and filtered by a 5–5,000 Hz bandpass using a Neuropack X1 (Nihon Kohden, Tokyo, Japan).

PMCT was calculated using the following formula: (F + M − 1)/2 (11). CMCT was calculated by subtracting PMCT from the shortest onset latency of MEP elicited by TMS (11). CECT was calculated by subtracting the shortest onset latency of MEP elicited by the magnetic stimulation over the first sacral (S1) vertebra from PMCT (12). The maximum amplitude ratio of MEP to CMAP (MEP/Mamp) was also evaluated.

#### 2.1.2 Somatosensory evoked potentials

Tibial somatosensory evoked potentials (SEPs) were recorded to assess the dorsal column-medial lemniscus. The tibial nerve on each side was stimulated at the ankle at a rate of 3 Hz using felt pad surface electrodes with 25 mm anode-to-cathode distance. The stimulus intensity was set at ≥150% of the motor threshold, the lowest stimulus intensity producing a twitch of the whole plantar muscles. The stimulus duration of the square pulse was 0.2 ms. Ag-AgCl surface cup recording electrodes were placed at the spinous process of the 12th thoracic vertebra (Th12S), iliac crest contralateral to the stimulation (ICc), Fz, and 2 cm posterior to Cz (Cz’) in the international 10–20 system. The evoked potentials were amplified and filtered by a 3–3,000 Hz bandpass, and 500 to 1,000 responses were averaged using a Neuropack X1. At least two averages were obtained to confirm the reproducibility of the evoked potentials. The measured parameters included the peak latency of N21 in the Th12S-ICc leads, the peak latency of P38 in the Cz’-Fz lead (ms), and central conduction time (CCT) from N21 to P38 (ms) (13).

### 2.2 Statistical analysis

Data are expressed as medians and interquartile ranges (IQRs). The Mann–Whitney U and Wilcoxon signed-rank tests were used for comparisons between independent groups and paired comparisons (before and after treatment), respectively. *p* values <0.05 were considered statistically significant.

## 3 Results

The median age at symptom onset was 51.0 (47.0–55.5) years, and baseline evaluations were performed at a median age of 66.7 (60.3–71.8) years. Male patients comprised 92% of the study cohort. No significant differences in age, height, or motor function were observed between the CA and NC groups. The demographic characteristics were similar between the SS and MS groups, whereas the median age of the MS group was significantly younger (Supplementary Table 1).

Abnormalities in MEP latencies and prolonged central motor conduction time (CMCT) were observed in 67 and 92% of patients with SS, respectively (Table 1).

**Table 1:** Assessments of central motor and sensory pathways in patients with superficial siderosis.

Parameter	Median (IQR)	No. of patients with abnormal values/total (*n*)	No. of patients with abnormal values/total (%)
MEP disappearance		1/12	8
F wave latency, ms	50.6 (46.6–54.3)	6/12	50
MEP latency, ms	43.8 (41.0–47.2)	8/12	67
MEP CMCT, ms	16.7 (15.5–18.3)	11/12	92
SEP P38 disappearance		0/12	0
SEP N21 latency, ms	22.9 (22.5–23.7)	0/11	0
SEP P38 latency, ms	44.1 (43.4–45.4)	7/12	58
SEP N21–P38 CCT, ms	20.8 (20.1–22.6)	11/11	100

MEP, motor evoked potential; CMCT, central motor conduction time; SEP, somatosensory evoked potential; CCT, central conduction time.

Abnormalities in MEP parameters (e.g., latency, CMCT, F wave) were defined according to reference (9). The normal limit values for SEP parameters (e.g., N21, P38, CCT) were based on a reference (11).

Although CMCT values exceeded the normal limits in both the SS and MS groups, significantly longer CMCT and MEP latencies were observed in the MS group (Table 2). Moreover, MEP amplitude (MEP amp) and the amplitude ratio of MEP to CMAP (MEP/Mamp) were significantly better maintained in the SS group. The rMT was significantly lower in the SS group compared with that in the MS group. CECT findings were within normal limits in both groups.

**Table 2:** Baseline electrophysiological characteristics of patients with superficial siderosis and multiple sclerosis.

Parameter	SS (*n* = 12)	MS control (*n* = 9)	*p-*value	Normal range	+2SD Upper limit of normal^#^
MEP appearance rate, No. %	11 (92)	7 (78)	0.38		
MEP latency, ms	43.8 (41.0–47.2)	49.6 (43.0–51.6)	0.03*	36.5 ± 2.3	42.3
CMCT, ms	16.7 (15.5–18.3)	23.9 (16.9–26.6)	0.03*	11.9 ± 1.3	15.2
F wave latency, ms	50.6 (46.6–54.3)	47.9 (46.1–50.2)	0.12	45.2 ± 2.3	51.0
PMCT, ms	26.6 (24.6–28.6)	25.1 (24.4–26.4)	0.09		
CECT, ms	3.7 (3.3–4.7)	3.9 (3.5–4.4)	0.96	3.4 ± 0.8	5.4
Distal motor latency, ms	3.8 (3.5–4.3)	3.6 (3.3–4.1)	0.23		
MEP amp, mV	3.2 (1.2–4.5)	0.5 (0.3–1.2)	0.002**		
M amp, mV	19.5 (15.9–23.4)	20.1 (13.2–26.3)	0.78		
MEP/M amp, %	18.8 (6.9–24.3)	1.7 (1.2–5.3)	0.002**		
rMT, %	78.0 (65.0–95.0)	100.0 (80.0–100.0)	0.02*		

SS, superficial siderosis; MS, multiple sclerosis; MEP, motor evoked potential; CMCT, central motor conduction time; PMCT, peripheral motor conduction time; CECT, cauda equina conduction time; MEP amp, MEP amplitude; M amp, muscle action potential amplitude; MEP/Mamp, amplitude ratio of MEP to compound muscle action potential; rMT, resting motor threshold; SD, standard deviation.

Values are median (IQR). *p < 0.05, **p < 0.01. Normal ranges are presented as mean ± SD. ^#^Normal ranges and upper limit of normal (mean + 2SD) for MEP latency, CMCT, and F wave latency are based on the data from reference (9). Normal ranges and upper limit of normal (mean + 2SD) for CECT are based on the data from a reference (10).

In the SEP examinations, prolonged latencies or absence of P38 were observed in 58% of patients with SS. All patients with measurable N21–P38 CCT demonstrated significantly prolonged conduction times (Table 1). The N21 latencies were preserved.

Baseline MEP parameters did not differ between the CA and NC groups. Administration of the iron chelator led to a significant improvement in the CA group after 36 months of treatment (Table 3). PMCT did not change significantly in either group. Interestingly, one patient who had no measurable MEP at the baseline regained detectable responses following the intervention (Supplementary Figure 2). In contrast, no significant changes were observed in SEP parameters in either group (Table 4).

**Table 3:** Transcranial magnetic stimulation parameters before and after iron chelator administration.

Parameter	CA group (*n* = 7)		NC group (*n* = 4)		Between-group *p*-values
	Median (IQR)	*p-*value; Baseline vs. F/U	Median (IQR)	*p-*value; Baseline vs. F/U	
MEP latency, ms					
Baseline	41.6 (40.5 to 45.3)	0.07	46.7 (44.8 to 48.1)	0.13	0.15
F/U	40.8 (38.2 to 44.2)		45.9 (43.7 to 47.7)		0.16
Difference	−1.4 (−1.7 to −0.6)		−0.7 (−0.9 to −0.4)		0.44
CMCT, ms					
Baseline	16.5 (15.7 to 17.3)	0.04*	17.2 (17.0 to 17.7)	0.88	0.32
F/U	14.6 (13.4 to 16.1)		17.5 (17.1 to 17.9)		0.11
Difference	−1.9 (−2.1 to −0.7)		+0.3 (−0.2 to +0.6)		0.04*
PMCT, ms					
Baseline	26.1 (24.3 to 26.8)	0.58	28.7 (27.5 to 30.0)	0.25	0.11
F/U	25.9 (24.8 to 27.1)		27.6 (26.6 to 29.0)		0.23
Difference	0.15 (−1.8 to +0.5)		−1.0 (−0.6 to −1.1)		0.07
CECT, ms					
Baseline	3.6 (3.1 to 4.3)	0.44	3.7 (3.5 to 4.2)	1.0	0.79
F/U	3.8 (3.2 to 5.1)		3.8 (3.4 to 4.3)		0.79
Difference	+0.3 (−0.0 to +0.9)		+0.0 (−0.5 to +0.3)		0.39
MEP amp, mV					
Baseline	2.5 (1.0 to 4.1)	0.95	2.9 (1.9 to 4.1)	0.88	0.93
F/U	3.1 (1.5 to 5.3)		2.6 (1.7 to 3.3)		0.57
Difference	+1.1 (−0.7 to +1.4)		+0.2 (−0.9 to +0.3)		0.32
MEP/M amp, %					
Baseline	14.6 (5.4 to 18.0)	0.31	23.3 (17.6 to 26.9)	0.63	0.28
F/U	15.3 (10.1 to 23.6)		15.8 (12.0 to 18.8)		0.93
Difference	+2.9 (−3.5 to +7.9)		−2.9 (−9.7 to +0.7)		0.12
rMT, %					
Baseline	84.0 (67.5 to 92.5)	0.56	77.5 (66.1 to 90.6)	1.0	0.98
F/U	71.2 (63.1 to 90.6)		75.0 (73.8 to 81.2)		0.48
Difference	−5.0 (−13.8 to +3.8)		+3.8 (−4.3 to +8.9)		0.45

CA, iron chelator administration group; NC, non-administration control group; MEP, motor evoked potential; F/U, follow-up; Difference, the difference of values between baseline and F/U; CMCT, central motor conduction time; PMCT, peripheral motor conduction time; CECT, cauda equina conduction time; MEP amp, MEP amplitude; MEP/Mamp, amplitude ratio of MEP to compound muscle action potential; rMT, resting motor threshold; Values are median (IQR). *p < 0.05.

**Table 4:** Somatosensory evoked potential parameters before and after iron chelator administration.

	CA group (*n* = 7)		NC group (*n* = 4)		Between-group *p*-values
	Median (IQR)	*p*-value; Baseline vs. F/U	Median (IQR)	*p*-value; Baseline vs. F/U	
SEP N21 latency, ms					
Baseline	22.9 (22.0 to 23.0)	0.11	23.7 (23.2 to 24.7)	0.38	0.08
F/U	23.5 (22.8 to 24.8)		25.6 (24.1 to 26.5)		0.32
Difference	+1.1 (+0.5 to +1.6)		+1.2 (+0.3 to +1.8)		0.89
SEP P38 latency, ms					
Baseline	44.0 (42.3 to 45.4)	0.11	44.5 (44.0 to 45.7)	0.13	0.39
F/U	45.3 (43.5 to 46.8)		47.0 (45.3 to 48.6)		0.50
Difference	+1.4 (+0.2 to +1.9)		+1.5 (+1.1 to +2.1)		0.68
SEP N21–P38 CCT, ms					
Baseline	21.9 (20.5 to 22.6)	0.47	20.4 (19.9 to 21.6)	0.25	0.65
F/U	22.8 (20.5 to 23.4)		22.1 (21.3 to 23.0)		0.83
Difference	+0.7 (−0.4 to +1.1)		+1.1 (+0.3 to +1.7)		0.68

SEP, somatosensory evoked potential; CA, iron chelator administration group; NC, non-administration control group; F/U, follow-up; Difference, the difference of values between baseline and F/U; CCT, central conduction time; Values are median (IQR). *p < 0.05.

No adverse events were observed during or after the administration of the tests.

## 4 Discussion

In this study, all patients with SS exhibited prolonged central motor and/or sensory tract conduction time. These conduction abnormalities were partially recovered with chelator administration. The relatively preserved MEP amplitude suggests that the pathophysiological mechanism underlying the conduction abnormalities in SS is likely different from that of MS, a typical demyelinating inflammatory disease.

Prolonged CMCT in SS suggests corticospinal tract damage caused by hemosiderin deposition, which leads to ferroptosis, oxidative stress, and inflammation (2, 7, 8, 14). The relatively preserved MEP amplitudes observed in patients with SS suggest that fewer central motor neurons and axons are affected.

Although prolonged N21–P38 CCT and abnormal P38 latency in SEP indicate sensory conduction impairments in the central nervous system (CNS), the preserved N21 latency suggests selective involvement of later sensory components. These findings align with the TMS results and emphasize the progressive impact of hemosiderin deposition on the central conduction pathways.

Proximal peripheral nerve function was preserved in the SS group, as evidenced by the normal CECT values. Pathological studies have confirmed limited hemosiderin deposition in peripheral nerves, which is consistent with the unimpaired functional results from electrophysiology (1, 15).

The iron chelator partially improved the CMCT in the treated patients, suggesting that neural injury can be partially reversed by removing iron and other heme-degrading products. Notably, one patient who initially had no measurable MEP displayed detectable responses after the intervention, further supporting the hypothesis that the chelator enhanced motor cortical excitability and restored conduction. The chelators appear to confer these benefits by reducing iron levels in the CNS, which may alleviate oxidative stress and ferroptosis, thereby enhancing neuronal excitability (2, 7, 8). Although the chelator partially improved central motor conduction, no significant recovery was observed in somatosensory evoked potentials. This may be because the dorsal column–medial lemniscus pathway is anatomically disposed closer to the spinal cord surface than the corticospinal tract, making it more susceptible to chronic inflammation and oxidative stress caused by subpial hemosiderin deposition (1, 2, 15).

Oligodendrocytes are the primary iron storage cells in the CNS (16). Their inability to process excess iron can lead to oxidative stress, ferroptosis, and other free iron-related toxicities, disrupting axon–oligodendrocyte interactions (15, 17). Axon–oligodendrocyte exchange of metabolic and structural support is essential for maintaining axonal integrity and proper myelination; disruption of this interaction may exacerbate axonal dysfunction. Iron chelators may help protect neurons by mitigating these effects.

This study has some limitations. The small sample size, owing to the rarity of SS, limits the generalizability of the findings. Further, the regulatory restrictions on iron chelator dosing might have influenced the therapeutic outcomes. Future studies with larger cohorts and optimized dosing regimens are required to validate these findings and further explore the potential of iron chelation therapy in SS.

In conclusion, hemosiderin deposition in the CNS causes significantly prolonged conduction time. The impairment was partially recoverable by removing deposited iron.

## Data Availability

The original contributions presented in the study are included in the article/Supplementary material, further inquiries can be directed to the corresponding author.
