# Supplementary material for: Quantitative evaluation of iron chelator effects on central motor and sensory tracts in superficial siderosis
Source: Front Neurol. 2026 Feb 18;17:1734951. doi: 10.3389/fneur.2026.1734951 (PMC12956528; doi:10.3389/fneur.2026.1734951)
Supplement: Supplementary file 1 [file Table_1.DOCX]

**Supplementary Materials**

**Supplementary Table 1: Demographic and clinical characteristics of the participants**

**Supplementary Figure 1: Study flowchart**

**Supplementary Figure 2: Representative MEP Before and After Deferiprone Administration**

**Supplementary Table 1. Demographic and clinical characteristics of the participants**

|  | **CA (n=8)** | **NC (n=4)** | ***p*-value**  **CA vs. NC** | **SS overall (n=12)** | **MS control (n=9)** | ***p*-value**  **SS vs. MS** |
| --- | --- | --- | --- | --- | --- | --- |
| Sex, male/female | 7/1 | 4/0 | >0.99 | 11/1 | 6/3 | 0.27 |
| Age at admission, years | 66.5 (62.5–71.8) | 62.5 (58.0–70.0) | 0.80 | 66.7 (60.3–71.8) | 39.0 (37.0–54.0) | 0.01* |
| Height, cm | 164.0 (159.2–170.4) | 168.8 (166.3–171.7) | 0.40 | 167.1 (160.8–170.6) | 169.0 (160.0–175.0) | 0.75 |
| Pyramidal sign, No. (%)  Babinski reflex  Hyperreflexia of the lower extremities | 3 (38%)  5 (63%) | 2 (50%)  2 (50%) | >0.99  >0.99 | 5 (42%)  7 (58%) | 7 (78%)  8 (89%) | >0.99  >0.99 |
| MMT score of AH | 5 | 5 | >0.99 | 5 | 5 | >0.99 |
| Duration between disease onset and  dural defect closure, months | 140.2 (89.1–165.6) | 157.8 (46.3–272.4) | >0.99 | 140.3 (50.2–205.4) |  |  |
| Duration between disease onset and  administration of iron chelator, months | 152.2 (100.5–182.3) |  |  |  |  |  |
| Duration between disease onset and baseline MEP test, months | 146.3 (99.7–182.6) | 171.9 (49.3–294.9) | >0.99 | 146.3 (51.6–218.5) |  |  |
| Administration period of deferiprone | 34.8 (30.8–36.0) |  |  |  |  |  |
| Duration between baseline MEP and the following MEP test, months | 34.9 (31.5–36.2) | 17.4 (15.0–23.6) | 0.09 | 33.6 (18.9–36.1) |  |  |

*CA*, iron chelator administration group; *NC*, non-administration control group; *SS*, superficial siderosis; *MS*, multiple sclerosis; *MMT*, manual muscle test; *AH*, abductor hallucis muscle; *MEP*, motor evoked potential

Values represent median (IQR). Pyramidal signs and reflex findings are shown as number (%). MMT scores were 5 in all patients. **p* <0.05.

**Supplementary Figure 1: Study Flowchart**

Of 62 patients who visited our hospital with superficial siderosis, 12 underwent dural defect closure and consented to participate in the study. Patients were assigned to a chelator administration group (n = 8) or non-administration group (n = 4). Electrophysiological tests, including transcranial magnetic stimulation (TMS) and somatosensory evoked potential (SEP), were conducted at baseline and at follow-up (median 33.6 months). For comparison, patients with multiple sclerosis (n = 9) underwent TMS and SEP testing at baseline only. Statistical analysis was performed using the Mann–Whitney U test. Data are presented as medians and interquartile ranges (IQRs). This flowchart reflects key components of the trial checklist, including patient flow, intervention assignment, and timing of assessment.

**
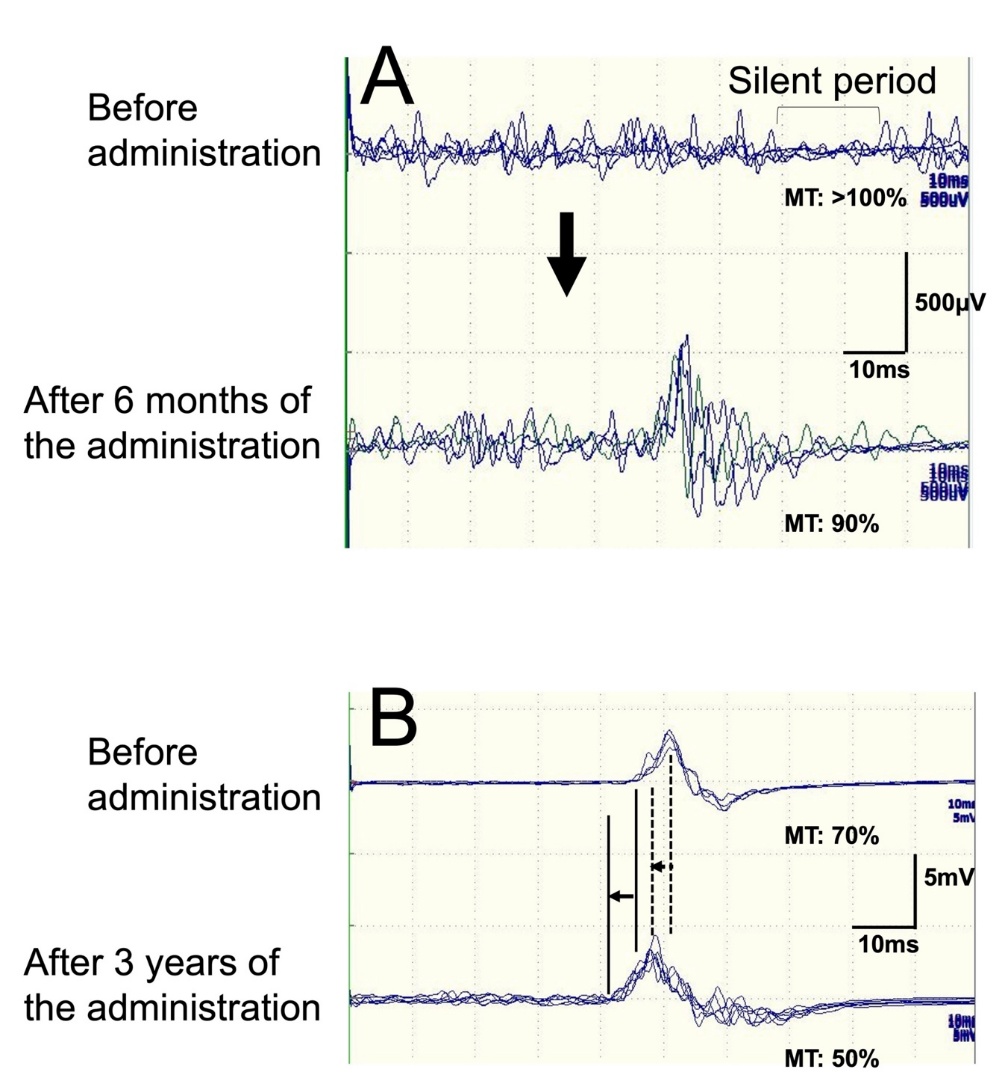
**

**Supplementary Figure 2: Representative MEP Before and After Deferiprone Administration**

Motor evoked potentials (MEPs) were elicited bilaterally with facilitation by slight contraction of the abductor hallucis (AH) muscle. (A) In one patient with superficial siderosis (SS), post-operative transcranial stimulation initially failed to elicit measurable MEPs. However, after 3 years of deferiprone treatment, distinct MEP was observed. (B) In another patient with SS, a significant reduction in the central motor conduction time (CMCT) was recorded after 3 years of deferiprone therapy. rMT, resting motor threshold
